# Single-Model Self-Recovering Fringe Projection Profilometry Absolute Phase Recovery Method Based on Deep Learning

**DOI:** 10.3390/s25051532

**Published:** 2025-03-01

**Authors:** Xu Li, Yihao Shen, Qifu Meng, Mingyi Xing, Qiushuang Zhang, Hualin Yang

**Affiliations:** 1 College of Electromechanical Engineering, Qingdao University of Science and Technology, Qingdao 266061, China; lx1041493745@163.com (X.L.);; 2 Hexagon Manufacturing Intelligence Technology (Qingdao) Co., Qingdao 266101, China

**Keywords:** fringe projection, absolute phase recovery, three-dimensional reconstruction, convolutional neural networks, deep learning

## Abstract

A drawback of fringe projection profilometry (FPP) is that it is still a challenge to perform efficient and accurate high-resolution absolute phase recovery with only a single measurement. This paper proposes a single-model self-recovering fringe projection absolute phase recovery method based on deep learning. The built Fringe Prediction Self-Recovering network converts a single fringe image acquired by a camera into four single mode self-recovering fringe images. A self-recovering algorithm is adopted to obtain wrapped phases and fringe grades, realizing high-resolution absolute phase recovery from only a single shot. Low-cost and efficient dataset preparation is realized by the constructed virtual measurement system. The fringe prediction network showed good robustness and generalization ability in experiments with multiple scenarios using different lighting conditions in both virtual and physical measurement systems. The absolute phase recovered MAE in the real physical measurement system was controlled to be 0.015 rad, and the reconstructed point cloud fitting RMSE was 0.02 mm. It was experimentally verified that the proposed method can achieve efficient and accurate absolute phase recovery under complex ambient lighting conditions. Compared with the existing methods, the method in this paper does not need the assistance of additional modes to process the high-resolution fringe images directly. Combining the deep learning technique with the self-recovering algorithm simplified the complex process of phase retrieval and phase unwrapping, and the proposed method is simpler and more efficient, which provides a reference for the fast, lightweight, and online detection of FPP.

## 1. Introduction

Optical measurement technology is widely used in intelligent manufacturing and industrial inspection due to its advantages of being non-contact, having high accuracy and efficiency, and suiting a wide range of applications [1]. FPP based on structured light has been proven to be one of the most effective techniques for obtaining three-dimensional (3D) shape information on objects [2,3,4,5,6]. This technique usually projects a fringe image with coded information onto the surface of an object, obtains a deformed fringe image that contains the modulation of the object’s contour information, and then decodes its phase to obtain the absolute phase, which can be combined with the principle of triangulation to compute the three-dimensional shape information of the object. Since the deformed fringe image involves inverse tangent calculation, the initial phase obtained will face the [−π, π] ambiguity problem, so two steps of phase retrieval and phase unwrapping are needed for phase decoding.

The traditional methods of phase resolution for FPP can be divided into Fourier-transform profilometry (FTP) [7,8,9,10,11] and phase-shifting profilometry (PSP) [12,13,14,15]. The phase map can be recovered from a single coded image using FTP. However, Fourier-transform-based methods smooth out steeply varying high-frequency information due to filtering operations performed in the spatial or time domain [16]. FTP is sensitive to noise and has a large phase resolution error for discontinuous objects. For the PSP method, three phase-shifted images are generally required, with high static measurement accuracy but low efficiency. Combining measurement accuracy and efficiency on standard equipment is challenging with traditional FPP methods. Data-driven deep learning technology provides more solutions for the FPP method [17,18]. Researchers have carried out a lot of research work in the field of fringe projection 3D measurement based on deep learning. It has been successfully applied to parcel phase solving, parcel phase unwrapping, fringe denoising, and fast 3D measurement, which initially realizes 3D measurement of a single fringe image [19,20,21].

Feng et al. [22], by analyzing the composition of the parcel phase, took the lead in adopting the prediction method of separating the numerator denominator terms of the parcel phase, and proposed a single-frame grating-fringe analysis method based on deep learning, which realizes phase demodulation by constructing two convolutional neural networks using only a single fringe image. It showed superior performance in terms of high accuracy and edge-preserving compared to two representative single-frame techniques: Fourier-transform profilometry [8] and windowed Fourier profilometry [9]. In order to further realize high-precision single-frame measurement of dynamic, high-speed moving objects, Feng et al. [23] introduced integrated learning technology on the basis of parcel phase numerator denominator term prediction, combining multiple models to work together as a substitute for a single neural network, proposing a K-fold average integration method to divide the dataset, and first adopting different datasets for the training of each basic network model. An adaptive integration neural network was used to fully automate and adaptively fuse the outputs of each base model, which effectively improved the prediction accuracy and generalization ability of the deep learning network model in the phase analysis task. Guo et al. [24] used a generalized framework to unify and simplify the temporal phase expansion, but it still required both low-frequency and high-frequency information for assisted retrieval.

At present, FPP still lacks efficient and accurate absolute phase recovery methods. The fringe order information computed by additional auxiliary modes is the reference basis for phase unwrapping of wrapped phases, and it is even more crucial to obtain high-precision full-field continuous absolute phase. However, it is still a challenge to perform efficient and accurate high-resolution absolute phase recovery with only a single measurement. To this end, researchers have proposed strategies for color coding and composite coding of structured light images [25,26,27,28]. Deep learning techniques also provide new solution ideas for color coding and composite coding strategies [29,30,31,32]. Qian et al. [29] encoded fringe patterns of three wavelengths into the red, green, and blue channels of a color image. A five-path convolutional neural network was designed to predict the wrapped phase arctan function numerator, denominator term, and coarse absolute phase. Absolute phase recovery was achieved by projecting a single-color fringe pattern. Li et al. [30] generated a kind of composite fringe by superimposing time domain information of different frequencies into the spatial domain based on the concept of spatial frequency multiplexing. A parallel U-Net network [31] was constructed for the prediction of wrapped phase and fringe order, respectively. Fu et al. [32] combined deep learning with binocular fringe projection. The three frequencies needed for the multi-frequency heterodyne method are passed through the three channels of the color image to form a single-composite-color fringe pattern, which achieves absolute phase acquisition from a single fringe image. However, the strategy of color coding has lower measurement accuracy for colored objects due to the object’s own color interference, and the composite coded image also introduces more complex phase encoding and decoding calculations.

Nguyen et al. [33] proposed a single-input dual-output network that contains one encoding path and two decoding paths to convert a single-shot image into two outputs: a multistep phase-shift image and a coarse phase map. Tan et al. [34] encoded a single fringe image acquired by a binocular system combined with a reference plane and a fringe order into a 5-channel input. A Y-shaped network was proposed to predict the wrapped phase and fringe order using two coding paths, respectively. However, the strategy of a multi-output path network is prone to limiting the final prediction accuracy due to the different task objects. The wrapped phase is directly predicted due to properties such as phase truncation, which can lead to interference in the learning of the neural network.

With deep learning’s end-to-end nature, Jeught et al. [35] trained a full convolutional neural network to accurately predict randomly generated height maps by simulating the generation of height maps on a large number of deformed fringe patterns. Nguyen et al. [36] found that by comparing three convolutional neural networks, U-Net performs the best in predicting depth by virtue of the symmetric structure and feature splicing of its encoding and decoding layers. Song et al. [37] proposed direct phase prediction from a single fringe image. An attention neural network based on deformation convolution was constructed, which mixed the deformation extraction stage and depth mapping stage and could directly convert the fringe image into the phase difference. This strategy is simple and efficient, but the neural network is difficult to fit accurately.

In order to realize the prediction of high-resolution data directly, Zhao et al. [38] designed a detailed restoration and super-reconstruction network to realize the transformation of the original low-resolution image into a high-resolution image directly. Wang et al. [39] realized high-resolution phase unwrapping by splicing the phase information and proposed a phase correction method to correct the spliced phase information. Li et al. [40] used patch synthesis of sinusoidal quantities of the phase information to realize dataset expansion and high-resolution prediction. In addition, directly changing the resolution of the deep learning network training dataset can also realize higher-resolution data prediction. However, the parameters of the corresponding network model will grow dramatically, resulting in more computational resources.

Most of the existing deep learning-based FPP methods are based on multiple fringe patterns, which makes network prediction or dataset selection more complicated when absolute phase recovery is performed directly. In order to realize accurate absolute phase recovery using only a single fringe pattern and inspired by a single mode self-recovering phase method [41], this paper proposes a single-model self-recovering fringe projection absolute phase recovery method based on deep learning. The constructed fringe prediction self-recovering network (FPSR-Net) can transform a single-fringe image into four-fringe images in self-recovering phase mode. After a simple self-recovering phase algorithm, efficient and robust absolute phase recovery from a single-fringe image is realized. At the same time, this paper takes the original fringe image with 8-bit depth as the learning object of the neural network model and adopts patch segmentation of the fringe image to realize the recovery of high-resolution absolute phase. The main contributions of this paper are as follows:

1. A single fringe image is transformed into four self-recovering fringe images by the proposed FPSR-Net. The network simultaneously enhances the feature extraction ability of fringe information from multiple scales, such as global and local, and has high prediction accuracy for fringe images;
2. Adoption of the fringe image splicing strategy to realize the prediction of high-resolution images. The original collected high-resolution images are segmented into multiple images identical to the training data by patch and transformed into four corresponding self-recovering phase fringe images by FPSR-Net. Subsequently, the network prediction results are merged into four original-resolution self-recovering fringe images. The wrapped phase, fringe order, and absolute phase are obtained by the single mode self-recovering algorithm;
3. The proposed method is trained based on the virtual dataset, and the pixel-by-pixel prediction error percentage of the predicted synthetic images is 0.1075–0.2703%. The absolute phase recovery error is only 0.015 rad for fringe images under different lighting conditions in the test set, and excellent performance can be achieved in real measurement scenarios;
4. Compared with previous deep learning methods, the method in this paper improves the single mode self-recovering phase algorithm, simplifies the absolute phase recovery process, and realizes efficient and accurate absolute phase recovery directly from a single fringe image without the aid of additional modes.

## 2. Materials and Methods

### 2.1. Principe of FPP Technique

The pixel-by-pixel light intensity $I_{i}$ in the N-step phase-shifted fringe image projected by the general temporal phase unwrapping can be expressed as Equation (1),

(1) $$I_{i}=A+Bcos(\Phi +2\pi i/N),$$

where *A* is the average intensity relating to the pattern brightness; *B* is the intensity modulation relating to the pattern contrast and surface reflectivity, Φ is the absolute phase and *i* is the number of phase-shift steps, i∈[0, N−1].

The single mode self-recovering phase method encodes the phase and the fringe order together into four fringe images in a single mode by embedding the fringe order into the phase-shift domain. The fringe pattern can be denoted as Equations (2)–(5).

(2) $$I_{1}^{P}\left(x,y\right)=A\left(x,y\right)+B\left(x,y\right)\sin\left[\Phi \left(x,y\right)+\alpha (x,y)\right],$$

(3) $$I_{2}^{P}\left(x,y\right)=A\left(x,y\right)-B\left(x,y\right)\sin\left[\Phi \left(x,y\right)-\alpha (x,y)\right],$$

(4) $$I_{3}^{P}\left(x,y\right)=A\left(x,y\right)-B\left(x,y\right)\cos\left[\Phi \left(x,y\right)+\alpha \left(x,y\right)\right],$$

(5) $$I_{4}^{P}\left(x,y\right)=A\left(x,y\right)+B\left(x,y\right)\cos\left[\Phi \left(x,y\right)-\alpha (x,y)\right],$$

where superscript *P* represents the projection image, $I_{n}^{P}\left(x,y\right)$ represents the intensity value of pixel $\left(x,y\right)$ in the fringe images, n∈[0,3]. Suppose *α* is the phase-shift term: $\Phi \left(x,y\right)$ is absolute phase which can be expressed as Equations (6) and (7),

(6) $$\Phi \left(x,y\right)=\frac{2\pi }{T}x,$$

(7) $$\alpha \left(x,y\right)=-\frac{\pi }{2}+\frac{\pi }{f}k(x,y),$$

where *T* is the fringe spacing, representing the number of pixels in a cycle, *f* is the fringe frequency, *k*(x,y) represents the fringe order, which encoded as k=int(x/T), *int* represents the truncated rounding function, and *x* represents the pixel position. Correspondingly, the light intensity $I_{i}^{C}\left(x,y\right)$ at pixel $\left(x,y\right)$ in the deformed fringe images captured by the camera is denoted sequentially as Equations (8)–(11),

(8) $$I_{1}^{C}\left(x,y\right)=A\prime \left(x,y\right)+B\prime \left(x,y\right)\sin\left[\Phi \left(x,y\right)+\alpha (x,y)\right],$$

(9) $$I_{2}^{C}\left(x,y\right)=A\prime \left(x,y\right)-B\prime \left(x,y\right)\sin\left[\Phi \left(x,y\right)-\alpha (x,y)\right],$$

(10) $$I_{3}^{C}\left(x,y\right)=A\prime \left(x,y\right)-B\prime \left(x,y\right)\cos\left[\Phi \left(x,y\right)+\alpha \left(x,y\right)\right],$$

(11) $$I_{4}^{C}\left(x,y\right)=A\prime \left(x,y\right)+B\prime \left(x,y\right)\cos\left[\Phi \left(x,y\right)-\alpha (x,y)\right],$$

where the superscript *C* represents the camera captured image. The average light intensity $A\prime \left(x,y\right)$ and the fringe modulation regime $B\prime \left(x,y\right)$ are related to the measurement environment and the surface material of the measured object. Thus, the wrapped phase $\Phi_{w}$ and the phase-shift term *α* can be obtained from Equations (12) and (13).

(12) $$\Phi_{w}\left(x,y\right)={tan}^{-1}[\frac{I_{1}^{C}\left(x,y\right)-I_{2}^{C}\left(x,y\right)}{I_{4}^{C}\left(x,y\right)-I_{3}^{C}\left(x,y\right)}],$$

(13) $$\alpha \left(x,y\right)={tan}^{-1}[\frac{I_{1}^{C}\left(x,y\right)+I_{2}^{C}\left(x,y\right)-I_{3}^{C}\left(x,y\right)-I_{4}^{C}\left(x,y\right)}{{-I}_{1}^{C}\left(x,y\right)+I_{2}^{C}\left(x,y\right)-I_{3}^{C}\left(x,y\right)+I_{4}^{C}\left(x,y\right)}],$$

However, the phase-shift term $\alpha \left(x,y\right)$ is solved with a spike error at the corresponding position when $I_{1}^{C}\left(x,y\right)=I_{4}^{C}\left(x,y\right)$ and $I_{2}^{C}\left(x,y\right)=I_{3}^{C}\left(x,y\right)$ occur simultaneously. This spike error must be removed using median filtering, and the result is expressed as $\alpha \prime \left(x,y\right)$ after filter correction. *Round* denotes the rounding function. The fringe order *k* can be obtained from Equation (14). Finally, the wrapped phase $\Phi_{w}\left(x,y\right)$ is expanded by Equation (15) into the single-cycle unwrapped absolute phase $\Phi \left(x,y\right)$ based on the decoded fringe order $k\left(x,y\right)$.

(14) $$k\left(x,y\right)=Round\{\left[\alpha \prime \left(x,y\right)+\frac{\pi }{2}\right]\times \frac{f}{\pi }\},$$

(15) $$\Phi \left(x,y\right)=\Phi_{w}\left(x,y\right)+2\pi k\left(x,y\right),$$

**Figure 1:**
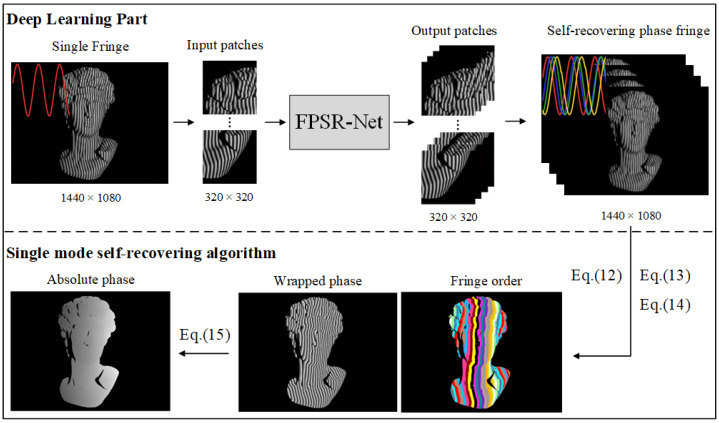
Flow diagram for the single-model self-recovering phase fringe projection technique using FPSR-Net for absolute phase recovery (Colored lines in deep learning part represent the trend of light intensity distribution in fringe images).

The overall flow of the single-model self-recovering fringe projection absolute phase recovery method is shown in Figure 1. Firstly, the acquired single high-resolution image is patch-segmented, which is used as the input of the FPSR-Net. The trained network outputs four fringe images of the single mode self-recovering phase method and re-stitches them into corresponding high-resolution images. The wrapped phase and fringe order are obtained by the self-recovering phase algorithm to realize efficient and accurate absolute phase recovery.

### 2.2. Fringe Prediction Self-Recovering Network Architecture

**Figure 2:**
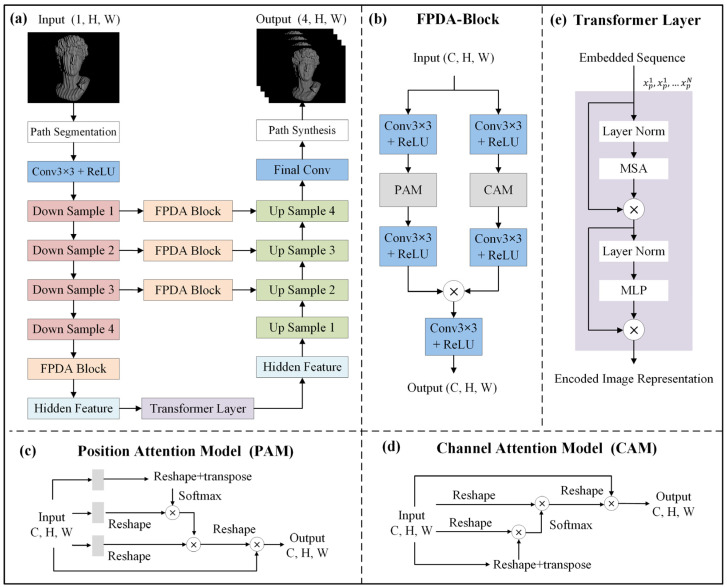
Fringe prediction self-recovering phase network. (**a**) Overall network architecture; (**b**) fringe prediction dual attention block; (**c**) position attention model; (**d**) channel attention model; (**e**) single transformer layer.

The network architecture of the proposed FPSR-Net is shown in Figure 2, which refers to DA-TransUNet [42], by introducing a transformer layer [43] and fringe prediction double attention block (FPDA-Block) into the U-Net network. The feature extraction ability of fringe information is enhanced from a global, local, and multi-scale perspective at the same time, which provides high prediction accuracy for fringe images. A single fringe image is encoded into a tensor of shape (C, H, W) as the input to the network, where (H, W) denotes the resolution of the fringe image and C represents the number of feature channels. For the input single fringe image, C = 1, and for the output four fringe images, C = 4. As shown in Figure 2a, the network input is subjected to the encoder for the three-layer down sampling convolution for the local feature extraction, where the convolutional kernel size is 3 × 3 and the convolution step is 1. The resolution of the feature map is halved after each down sampling convolution. The feature channels are 64, 128, 256, and 512 in turn. The back section of the encoder is optimized by the FPDA-Block, as shown in Figure 2b, so that the features are more suitable for the transformer layer. After dimensional compression, the global features are extracted by the transformer layer without changing the size of the feature map, as shown in Figure 2e. The output of the transformer layer is reconverted to a 3D feature map after dimensional reshaping and used as an input to the decoding layer. A skip connection consisting of FPDA-Block at three different scales of the encoder splices the encoder with the decoder for feature splicing. The decoder performs layer-by-layer convolution and up-sampling to achieve multi-scale feature fusion. Four channels are finally generated by the convolutional layer at the end of the decoder to output the corresponding fringe images.

The adopted FPDA-Block (Figure 2b) is integrated from both the position and channel perspectives of feature extraction to enhance the accuracy and granularity of features. As a result, more accurate and detailed features can be obtained in the encoder and in the jump connection, thus enhancing the prediction ability of the network model. The dual attention module is mainly divided into position attention module (PAM, Figure 2c) and channel attention module (CAM, Figure 2d). The PAM is used to extract the spatial dependence of different positions of the fringed image in the local feature map, taking the feature similarity between any two positions as the weights, updating the specific features at different positions by weighted summation, and mainly dealing with the two dimensions of the feature map. The CAM is used to extract the mutual influence relationship of the feature maps in different channels of the fringe image, adopting the degree of influence between any two channels as the weight, and updating the specific features of each channel by means of weighted summation as well, mainly dealing with the feature channel dimension of the feature map.

## 3. Experiment Results

### 3.1. Virtual Measurement System Construction and Datasets Preparation

**Figure 3:**
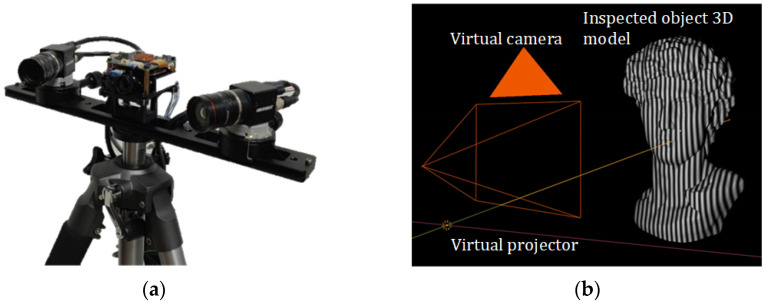
Schematic diagram of measurement system. (**a**) Physical measurement system (only one camera was used in this paper); (**b**) simulated measurement system.

Adequate training data guarantee good results for deep learning. The data acquisition of the actual measurement system is more time-consuming, and the system calibration parameters relied on in the reconstruction process are random in nature, which is not suitable for batch dataset preparation and actual deployment. In this paper, the open-source 3D modeling software Blender 4.0 was selected to build a fringe projection virtual measurement system based on the actual measurement system [44], and the calibration parameters of the actual physical measurement system were used as inputs to the virtual measurement system. The single mode self-recovering phase algorithm described in Section 2.1 was used to prepare the dataset in the virtual measurement system. The actual measurement system contained a DLP 3010 digital projector (Texas Instruments, Dallas, TX, USA, 1280 × 720 pixels) and an MV-CA016-10UM camera (Hikvision, Hangzhou, China, 1440 × 1080 pixels). The parameters of the actual measurement system are shown in Table 1. The building scene of the physical measurement system and the virtual measurement system are shown in Figure 3.

In contrast to Zhu et al. [45], who directly used the virtual measurement system to obtain the depth mapping of the measured object, the virtual measurement system constructed in this paper was used to obtain the modulation information of the fringe image on the surface of the measured object. Since the camera and projector in the virtual measurement system are ideal models for simulation, in order to minimize the gap between the fringe image rendered in the virtual measurement system and the actual measurement image, the training results of the virtual measurement system dataset were guaranteed to be directly applied to the real measurement scene. In this paper, we added aberration parameters to the nodes and renderer of the virtual projector based on Zhang et al. [46] and Wang et al. [47] to realize the internal parameter matching between the physical measurement system and the virtual measurements. Using the camera calibration based on 2D planar target and the “camera-projector” calibration based on phase shifting [48,49,50], the comparison of the calibration results of the physical measurement system and the virtual measurement system’s calibration parameters of the internal and external parameters are shown in Table 2 and Table 3.

**Figure 4:**
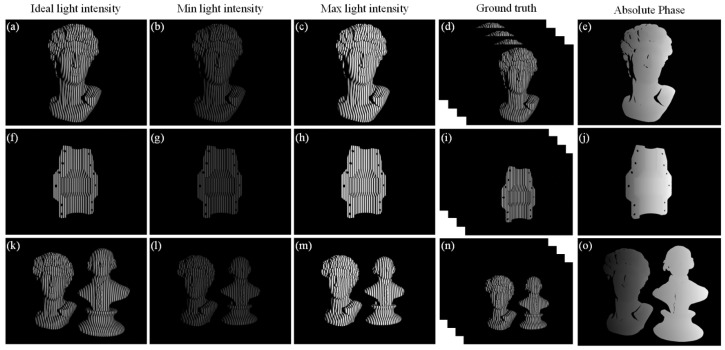
Example sets of three scenarios in the dataset: (**a**,**f**,**k**) ideal light intensity; (**b**,**g**,**l**) minimum light intensity; (**c**,**h**,**m**) maximum light intensity; (**d**,**i**,**n**) ground truth; (**e**,**j**,**o**) absolute phase of ground truth recovery.

To ensure the generalization of the network, 300 3D models of arbitrary categories were selected as the objects to be measured. The rendering process first introduced the model of the object to be measured to be placed in the measurement field of view and perform random bit position changes. After each change, four fringe images under ideal light intensity were first rendered as ground truth. Subsequently, to improve the robustness of this method against ambient light, the projected light intensity was set to vary randomly in the range of 5 W−60 W to simulate ambient light interference, and a single fringe image was rendered as the network input. All the operations in the virtual measurement system were performed automatically based on the Python 3.8 interface, which can greatly improve the efficiency of dataset preparation. The modulation range of the fringe image under ambient light interference was 18.6840–121.2425, and the modulation range of the ground truth fringe image under ideal light intensity was 54.7142–65.6696. There were 2500 sets of 320 × 320 pixels datasets prepared by windowing, and the ratio of training, validation, and test sets was 8:1:1. Figure 4 shows the example sets of three scenarios in the test set.

### 3.2. Patch Image Synthesis Analysis

**Figure 5:**
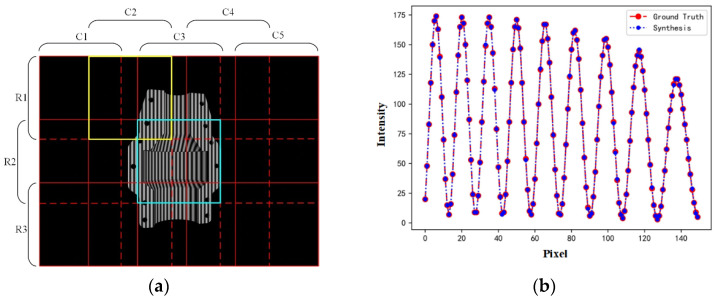
Image path segmentation, synthesis, and comparison. (**a**) Schematic of image segmentation and synthesis (The red solid line is the start position of each patch block segmentation, and the red dashed line is the end position. The yellow and blue areas represent the R1C2 patch and the R2C3 patch, respectively); (**b**) comparison of predicted results combined with ground truth.

The dataset needs to be cropped according to the input and output sizes set by the network before network training. The segmentation and synthesis of the image using patch are shown in Figure 5a. In order to ensure the continuity of fringes between the edges of the cropped patches, a certain overlapping area was required between neighboring patches. After network training, the prediction accuracy was lowest at the patch edges. Therefore, the predicted values at the patch edges were not involved in the image synthesis, and the predicted values of the neighboring patches were used directly in the patch edge region as the synthesis result. The overlapping region with patch edges removed adopts the average value of neighboring patch blocks as the synthesis result. Taking the upper left corner of the image as the origin, the red solid line is the starting position of each patch block segmentation, and the red dashed line is the termination position. R1C2 represents the yellow region patch and R2C3 represents the blue region patch. The synthesized images at randomly selected edge locations were analyzed with ground truth and the results are shown in Figure 5b. The synthesized images of predicted fringes were compared with the ground truth on a pixel-by-pixel basis, and the error percentage was 0.1075–0.2703%. The fringe continuity of the synthesized image was not different from the ground truth, and the difference in pixel-by-pixel position did not affect the subsequent dephasing process.

### 3.3. Network Implementation and Training

The dataset preparation and network training were deployed on the Dell Tower workstation, the Precision 7920, and 150 rounds of training iterations were performed on RTX 3090 GPUs (NVIDIA, Santa Clara, CA, USA, 24GAM) based on the Pytorch deep learning framework. The mini batch size was set to 4, the Adam Optimizer was used to optimize the network parameters, and the learning rate was initially set to 0.0001. CosineAnnealingLR was used to reduce the learning rate to 1 × 10^−6^ at 120 rounds and maintained thereafter.

**Figure 6:**
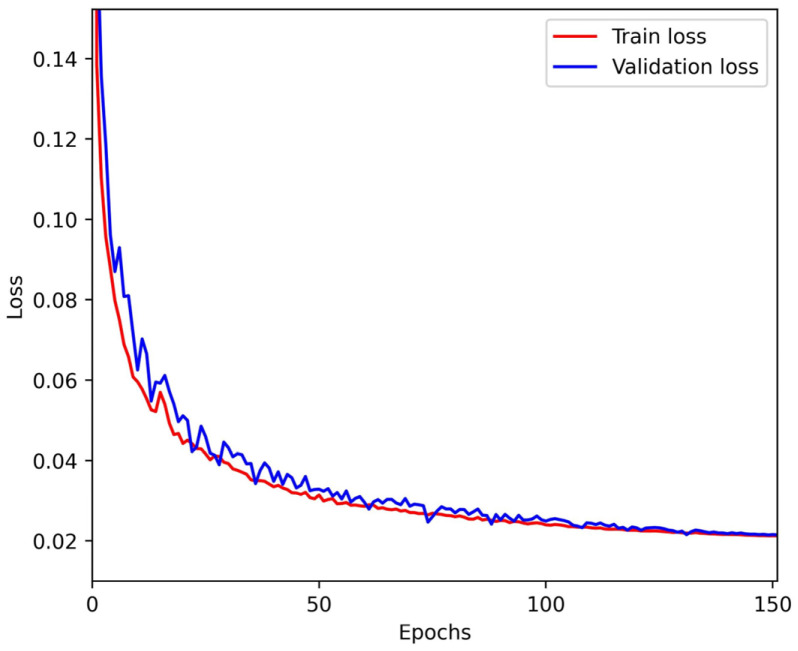
The loss curve of the training stage.

In contrast to N-Step phase-shifted images, the distribution of phase differences between single mode self-recovering fringe images is not regular. The loss function in this paper is a combination of image structural similarity, smoothing L1 loss, and the MSE of the foreground based on the threshold of the modulation [30]. The fringe prediction self-recovering phase network loss function is denoted as Equation (16),

(16) $$Loss=\lambda_{1}\left(1-SSIM\left({gt}_{i}-{pre}_{i}\right)\right)+\lambda_{2}L1({gt}_{i}-{pre}_{i})+\lambda_{3}MSE({gt}_{Bi}-{pre}_{Bi})$$

where *pre* represents the network prediction value, and *gt* represents the corresponding reference ground truth. SSIM represents the image structure similarity, L1 represents the smoothing L1 loss, and *MSE* represents the mean square error of the foreground computed according to the threshold of the modulation system. $\lambda_{1}$, $\lambda_{2}$, $\lambda_{3}$ represents the adjustable weights of each loss function. The results of the distribution of the loss function during training are shown in Figure 6, and the network converges after 120 rounds.

Table 4 shows the fringe prediction error (MAE between the network predicted value and the ground truth) of FPSR-Net when different loss functions were employed, as well as the MAE of the corresponding recovered absolute phase. For the self-recovering phase fringe images, both the fringe prediction error and the absolute phase error were largest when only MSE loss was used. The use of Smooth L1 improved the error results by a small margin compared to the use of MSE loss. Meanwhile, the introduction of SSIM effectively improved the overall prediction accuracy of the fringe images. The further addition of the foreground of the measured object localized by the threshold MSE of the modulation system effectively removed the interference of the background region and improved the prediction accuracy of the foreground.

### 3.4. Model Comparison Experiment

**Figure 7:**
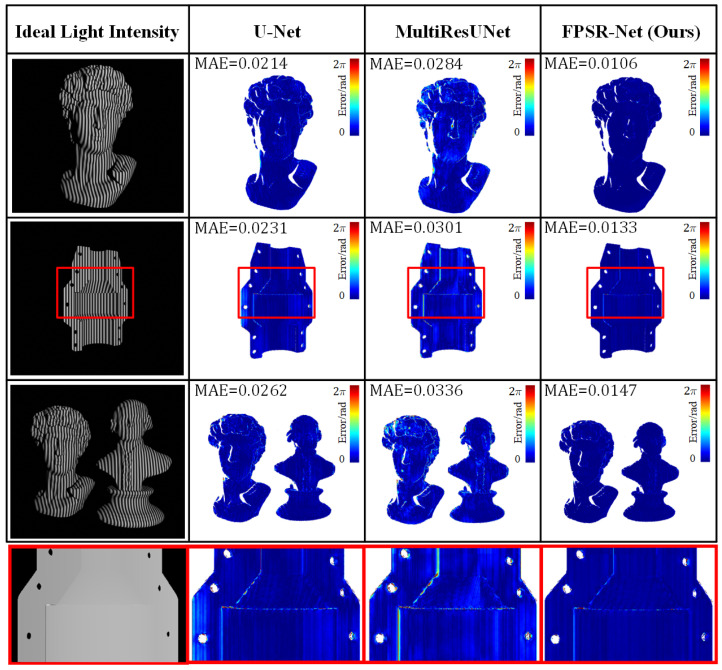
Absolute phase measurement error results for three scenarios (The red box represents a partial zoomed-in view).

Previously, the basic architecture network of U-Net was usually used for fringe image processing. To verify that FPSR-Net can improve the prediction accuracy of fringe images, this section compares the absolute phase recovery accuracy of U-Net, MultiResUNet, and the proposed FPSR-Net. The absolute phase information solved by the single mode self-recovering algorithm for four fringe images at an ideal light intensity of 25 W was used as the ground truth. A 5 W−60 W random ambient light interference was subsequently added based on the test set. Table 5 compares the absolute phase MAE recovered under different ambient light conditions by directly employing the single mode self-decomposition algorithm, the U-Net-based network, the MultiResUNet-based network, and the proposed FPSR-Net network. The absolute phase MAE recovered by using three network models for each of the three scenarios under ideal light intensity is shown in Figure 7.

It was found that the absolute phase recovered under ambient light interference by directly adopting the single mode self-recovering phase algorithm has a great error. The deep learning approach not only realizes absolute phase recovery from a single image but also effectively resists ambient light interference. U-Net, MultiResUNet [51], and FPSR-Net were compared among the deep learning-based methods. Among them, MultiResUNet had the largest error in absolute phase recovery, and the proposed FPSR-Net had the smallest error.

### 3.5. Ablation Experiment

**Figure 8:**
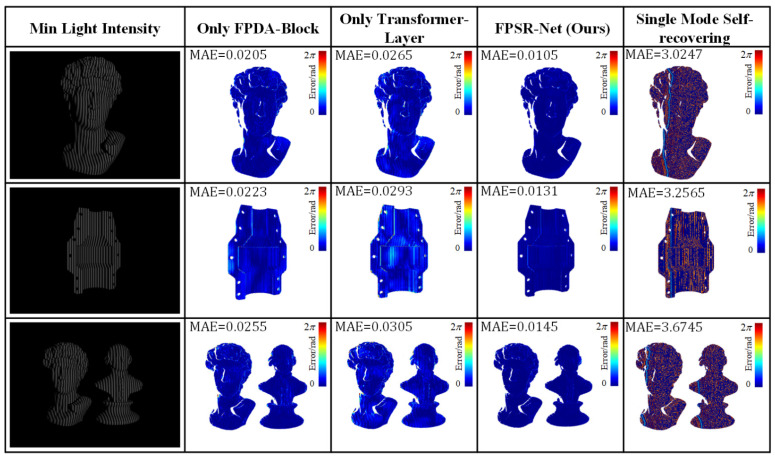
Absolute phase measurement error results for different modules under dim ambient light interference.

**Figure 9:**
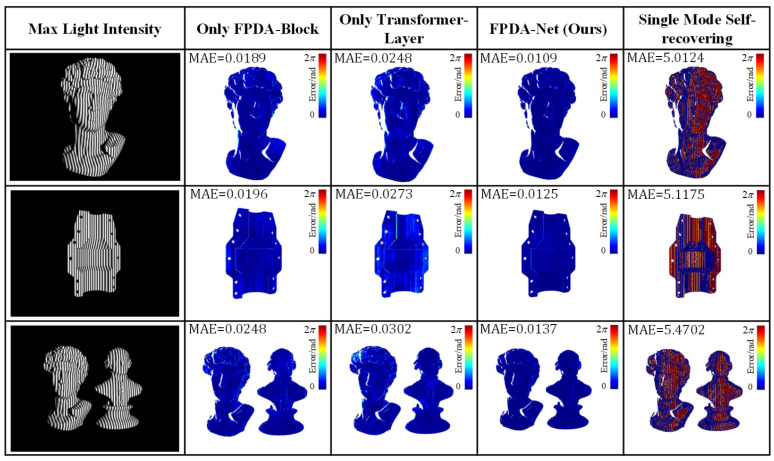
Absolute phase measurement error results for different modules under exposure ambient light interference.

In order to verify the ability of different modules in the FPSR-Net to contribute to the ambient light interference, only the FPDA-Block, the transformer layer, FPSR-Net, and the direct use of the single mode self-dephasing method were compared for the absolute phase restoration MAE under ambient light interference, respectively. Figure 8 demonstrates the absolute phase recovery results at a minimum light intensity of 5 W, and Figure 9 demonstrates the absolute phase recovery results at a maximum light intensity of 60 W.

As a benchmark comparison method, the direct use of the single mode self-recovering method had a great error when facing complex ambient light interference. When only the FPDA-Block was used, the absolute phase recovery error was smaller than that of the direct single mode self-recovering method. This shows that the FPDA-Block module can suppress the effect of ambient light and improve the accuracy of absolute phase recovery. When only the transformer layer is used for feature extraction and enhancement, its resistance to ambient light interference was better than that of the single mode self-phase decomposition method but not as good as that of the FPDA-Block, and when the FPDA-Block and transformer layer were combined, the combination of the two modules complemented each other, which made the FPSR-Net more effective in dealing with ambient light interference and able to achieve more accurate absolute phase recovery. 

As a benchmark comparison method, the direct application of the single-mode self-recovering technique exhibited significant errors when confronted with complex ambient light interference. In contrast, when utilizing only the FPDA-Block, the absolute phase recovery error was notably smaller than that observed with the direct single mode self-recovering approach. This indicates that the FPDA-Block module effectively mitigated the impact of ambient light and enhanced the accuracy of absolute phase recovery. When solely employing the transformer layer for feature extraction and enhancement, its resistance to ambient light interference surpassed that of the single-mode self-phase decomposition method; however, it did not perform as well as the FPDA-Block. Furthermore, when combining both FPDA-Block and transformer layer modules, their complementary strengths resulted in an FPSR-Net that was more adept at addressing ambient light interference and achieving greater precision in absolute phase recovery.

The results show that the FPDA-Block and the transformer layer in the FPSR-Net contributed significantly to improving the robustness of the system against ambient light interference. The two form the overall FPSR-Net architecture, which can provide optimal performance, proving the rationality and effectiveness of the FPSR-Net.

### 3.6. Actual Measurement System Reconstruction Results

**Figure 10:**
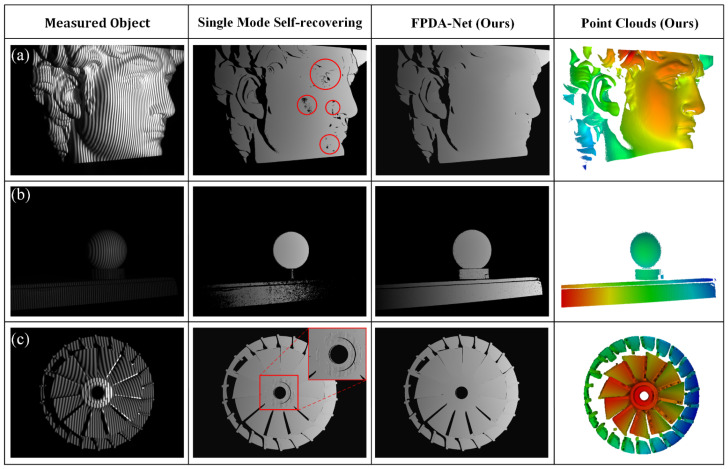
Measurement results under actual ambient light interference. (**a**) Exposure scene, (**b**) dim scene, (**c**) reflection scene. (The red color indicates the location of the phase defect).

Figure 10 shows the results of the trained FPSR-Net in a real measurement system. The absolute phase recovery results of FPSR-Net and the single mode self-recovering method were compared by three measurement scenarios, namely, exposure (Figure 10a), dim (Figure 10b), and reflection (Figure 10c), and the point cloud was reconstructed based on the absolute phase recovered by the FPSR-Net. Under non-ideal light intensity conditions, the absolute phases recovered directly using the single mode self-recovering algorithm were all defective to different degrees, as shown in the red-marked area in Figure 10. Under all three kinds of ambient light interference, the FPSR-Net obtained accurate absolute phase information, which reduced the information loss caused by ambient light interference. The reconstructed point cloud fitting RMSEs were 0.0015 mm, 0.0011 mm, and 0.0019 mm for exposure, dim, and reflection scenes, respectively. The experimental results show that the FPSR-Net is able to effectively cope with the ambient light interference in the real measurement system, maintain the high accuracy of the phase restoration, and improve the robustness of the measurement system, which verifies the effectiveness of the FPSR-Net in the practical measurement system.

**Figure 11:**
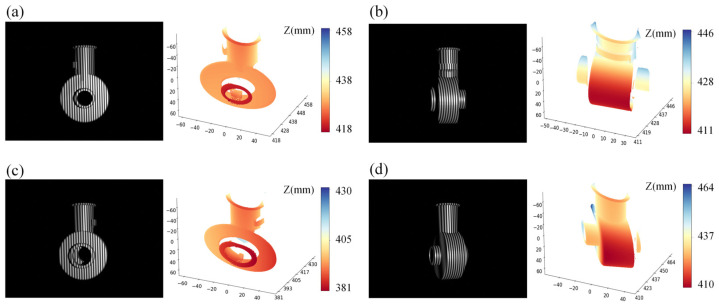
Measurement results of the dynamic scene. (**a**–**d**) Images and measurement results of proposed FPSR-Net at different times.

To evaluate the effectiveness of the proposed method in dynamic scenes, the object under test was placed on a motorized turntable to simulate the dynamic motion process. The real-time reconstruction results of the proposed FPSR-Net are demonstrated in a reflective scene. The captured images and measurement results of the dynamic scene are shown in Figure 11. The proposed FPSR-Net successfully reconstructed the 3D shape of the measured object, thus validating the effectiveness of the FPSR-Net in dynamic scenes.

## 4. Conclusions

In this paper, we show how to improve the single mode self-recovering phase method based on deep learning to realize efficient and accurate high-resolution absolute phase recovery relying only on a single fringe image. A fringe prediction self-recovering network was constructed for the fringe image features, which can transform a single fringe image acquired by the camera into four single mode self-recovering fringe images. The constructed virtual measurement system takes the calibration parameters of the physical measurement system as inputs and verifies the accuracy and reproducibility of the virtual measurement system against the physical measurement system, which simplifies the complicated data acquisition process. The trained FPSR-Net network can be directly applied to the real physical measurement system. Patch segmentation and synthesis are directly adopted to process the high-resolution fringed images, and the predicted synthesized images guarantee fringe continuity, while the pixel-by-pixel prediction error percentage is only 0.1075–0.2703%. According to the experimental verification, the FPSR-Net has strong robustness and generalization ability for different fringe patterns and different ambient light disturbances. The proposed method relies on a single fringe image to achieve efficient and accurate absolute phase recovery for high-resolution images. Applying the training results based on the virtual measurement system to the physical measurement system, the recovered absolute phase MAE was controlled to be 0.015 rad, and the point cloud reconstruction fitting RMSE was controlled to be 0.02 mm. Compared with the existing deep learning-based methods, the proposed method directly processes the high-resolution fringe image. In terms of phase retrieval and phase unwrapping, the FPSR-Net does not need the reference of additional information and only relies on a single-fringe image, which is simpler and more efficient.

## 5. Discussion

The method proposed in this paper focuses on the absolute phase recovery of general object surfaces and shows good performance in this type of scenario. However, it is important to note that this method does have certain limitations. The analysis of the limitations of this method and the improvement strategies will be given below to further guide future research based on this paper.

Firstly, for highly reflective or low-brightness surfaces, the contrast of the fringe image is susceptible to being affected, which can lead to some degradation of the phase recovery accuracy. Further optimization of the network model is considered, along with the addition of datasets targeting objects with highly reflective or low-brightness surfaces, using specific methods to prepare datasets. In addition, the applicability of this method can be enhanced by drawing on adaptive light adjustment techniques or developing optimization strategies for fringe coding on special surfaces.

Secondly, it was discovered that the absolute phase recovery error during the network’s prediction phase is concentrated in the object’s edge region. This phenomenon is challenging to fully avoid and has an impact on the accuracy of the reconstruction. A deep learning network module specifically applicable to fringe image features in FPP is considered to be proposed, strengthening the ability to capture key features in the edge region of the object. Constructing an edge composite loss function would guide the network to pay more attention to the information of the object edges and optimize the learning effect of the network on the object edges. Further in-depth study of the improvement strategies of traditional FPP methods is suggested in dealing with the error of edge regions.

In addition, the proposed FPSR-Net reduces four fringe images to single shots in terms of efficiency compared to the traditional method. However, the computational requirements of this network may somewhat limit its deployment in embedded or low-power environments. Consideration should be given to introducing a lightweight network architecture that reduces the computational effort and parameters through operations such as deeply separable convolution. Alternatively, model pruning, quantization techniques, and knowledge distillation methods can be used to reduce computational resource requirements while ensuring measurement accuracy.

In conclusion, deep learning technology provides more possibilities in the field of optical metrology, and this study provides a reference for realizing efficient and accurate FPP technology.

## Figures and Tables

**Table 1:** Physical system parameters.

Parameters	Value	Parameters	Value
Camera resolution	1440 × 1080 pixel	Aperture number F	8
Camera focal length	10 mm	Fringe pitches	14.6 pixel
Projector resolution	1280 × 720 pixel	Phase-shift steps	4
Light intensity of projector	5 W–60 W	Sample count per pixel	1024

**Table 2:** Comparison of internal parameter calibration results for physical and virtual measurement systems.

Internal Parameters	$\alpha_{x}$	$\alpha_{y}$	$u_{0}$	$v_{0}$	$k_{1}$	$k_{2}$	$p_{1}$	$p_{2}$
Real camera	2359.53	2359.09	734.47	578.34	−0.1251	0.1620	0.0003	−0.0004
Real projector	1700.15	1701.22	627.73	406.54	0.1328	−0.3361	−0.0006	−0.0001
Visual camera	2359.45	2359.07	734.38	578.27	−0.1237	0.1612	0.0003	−0.0004
Visual projector	1700.09	1701.21	627.75	406.57	0.1315	−0.3358	−0.0006	−0.0001

**Table 3:** Comparison of external parameter calibration results for physical and virtual measurement systems.

External Parameters	$\theta_{x}$	$\theta_{y}$	$\theta_{z}$	$t_{1}$	$t_{2}$	$t_{3}$
Physical system	−0.0099	0.3754	0.0276	−94.5914	−0.1889	12.4706
Visual system	−0.0099	0.3754	0.0276	−94.5914	−0.1889	12.4706

**Table 4:** Error comparison of different loss functions.

Loss Option	Fringe MAE	Absolute Phase MAE (Rad)
MSE	0.0079	0.0198
SmoothL1	0.0073	0.0168
SmoothL1 + SSIM	0.0064	0.0142
SmoothL1 + SSIM + BMSE	0.0052	0.0102

**Table 5:** Comparison of absolute phase recovery errors of different methods (MAE/rad).

Model Option	Ideal Light Intensity	Min Light Intensity	Max Light Intensity
Tradition		2.9677	5.1429
UNet(DL)	0.0241	0.0259	0.0241
MultiResUNet(DL)	0.0308	0.0367	0.0316
FPSR-Net(DL-Ours)	0.0102	0.0104	0.0110

## Data Availability

The original contributions presented in this study are included in the article. Further inquiries can be directed to the corresponding author.
